# 2-Hydroxy­imino-*N*′-[1-(2-pyrid­yl)ethyl­idene]propanohydrazide

**DOI:** 10.1107/S1600536809033352

**Published:** 2009-08-26

**Authors:** Yurii S. Moroz, Irina S. Konovalova, Turganbay S. Iskenderov, Svetlana V. Pavlova, Oleg V. Shishkin

**Affiliations:** a National Taras Shevchenko University, Department of Chemistry, Volodymyrska Str. 64, 01033 Kyiv, Ukraine; b SCT ‘Institute for Syngle Crystals’, National Acadamy of Science of Ukraine, Lenina Ave. 60, 61001 Kharkiv, Ukraine

## Abstract

The title compound, C_10_H_12_N_4_O_2_, features an intra­molecular N—H⋯N hydrogen bond formed between the imine NH and oxime N atoms. The oxime group and the amide C=O bond are *anti* to each other. In the crystal, mol­ecules are connected by O—H⋯O hydrogen bonds into supra­molecular zigzag chains along the *c* axis.

## Related literature

For oxime and pyridine derivatives, see: Sliva *et al.* (1997*b* ▶); Mokhir *et al.* (2002 ▶); Krämer *et al.* (2002 ▶); Kovbasyuk *et al.* (2004 ▶). For 2-hydroxy­imino­propanamide and amide derivatives of 2-hydroxy­imino­propanoic acid, see: Onindo *et al.* (1995 ▶); Duda *et al.* (1997 ▶); Sliva *et al.* (1997*a* ▶). For the preparation and characterization of 3*d*-metal complexes with 2-hydroxy­imino-*N*′-[1-(2-pyrid­yl)ethyl­idene]propano­hydra­zone, see: Moroz *et al.* (2008*a* ▶,*b* ▶). For typical bond lengths, see: Bürgi & Dunitz (1994 ▶).
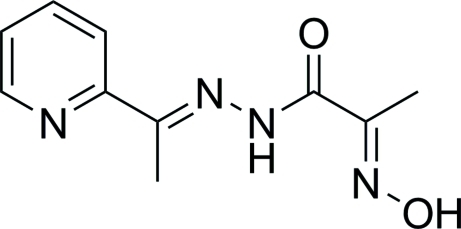


## Experimental

### 

#### Crystal data

C_10_H_12_N_4_O_2_
*M* *_r_* = 220.24
Monoclinic, 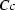

*a* = 4.4498 (11) Å
*b* = 22.833 (7) Å
*c* = 10.955 (3) Å
β = 97.47 (2)°
*V* = 1103.7 (5) Å^3^
*Z* = 4
Mo *K*α radiation
μ = 0.10 mm^−1^
*T* = 293 K
0.15 × 0.10 × 0.05 mm

#### Data collection

Oxford Diffraction KM-4/Xcalibur diffractometer with a Sapphire3 detector
Absorption correction: multi-scan (*CrysAlis CCD*; Oxford Diffraction, 2006 ▶) *T*_min_ = 0.986, *T*_max_ = 0.995
3899 measured reflections
958 independent reflections
793 reflections with *I* > 2σ(*I*)
*R*_int_ = 0.059

#### Refinement

*R*[*F* ^2^ > 2σ(*F* ^2^)] = 0.046
*wR*(*F* ^2^) = 0.097
*S* = 1.10
958 reflections
155 parameters
2 restraints
H atoms treated by a mixture of independent and constrained refinement
Δρ_max_ = 0.13 e Å^−3^
Δρ_min_ = −0.16 e Å^−3^

### 

Data collection: *CrysAlis CCD* (Oxford Diffraction, 2006 ▶); cell refinement: *CrysAlis RED* (Oxford Diffraction, 2006 ▶); data reduction: *CrysAlis RED*; program(s) used to solve structure: *SHELXS97* (Sheldrick, 2008 ▶); program(s) used to refine structure: *SHELXL97* (Sheldrick, 2008 ▶); molecular graphics: *ORTEP-3 for Windows* (Farrugia, 1997 ▶); software used to prepare material for publication: *WinGX* (Farrugia, 1999 ▶).

## Supplementary Material

Crystal structure: contains datablocks I, global. DOI: 10.1107/S1600536809033352/tk2523sup1.cif

Structure factors: contains datablocks I. DOI: 10.1107/S1600536809033352/tk2523Isup2.hkl

Additional supplementary materials:  crystallographic information; 3D view; checkCIF report

## Figures and Tables

**Table 1:** Hydrogen-bond geometry (Å, °)

*D*—H⋯*A*	*D*—H	H⋯*A*	*D*⋯*A*	*D*—H⋯*A*
O2—H2*OA*⋯O1^i^	0.84 (5)	1.88 (5)	2.709 (4)	170 (5)
N3—H3*NA*⋯N4	0.87 (4)	2.30 (4)	2.640 (4)	104 (3)

Symmetry code: (i) 
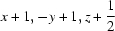
.

**Fig. 1.**
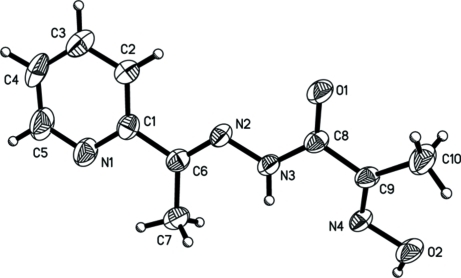
A view of (I), with displacement ellipsoids shown at the 40% probability level and atom labelling.

**Fig. 2.**
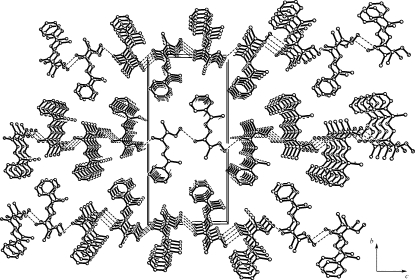
A packing diagram for (I) viewed in projection down the a axis. Hydrogen bonds are indicated by dashed lines; H atoms are omitted for clarity.

## supplementary crystallographic information

### Comment

As a part of our on-going work, we would like to report the structure of the title compound (I), Fig. 1, which comprises several groups capable of forming hydrogen bonding interactions: oxime, hydrazone, azomethine, and pyridine. Molecule (I) has been shown previously to form mono- and tetra-nuclear grid-like complexes with 3d-metals (Moroz *et al.*, 2008*a*,*b*).

The C—N and N—O bond lengths in the oxime group, i.e. 1.285 (5) and 1.388 (4) Å, respectively, adopt typical values (Sliva *et al.*, 1997*b*; Mokhir *et al.*, 2002). The oxime group is in an *anti*- position with respect to the amide group, an observation consistent with the structures of 2-hydroxyiminopropanamide and other amide derivatives of 2-hydroxyiminopropanoic acid (Onindo *et al.*, 1995; Duda *et al.*, 1997; Sliva *et al.*, 1997*a*). This conformation is stabilised by an N3—H···N4 intramolecular interaction, Table 1. The CH_3_C(=NOH)C(O)NH fragment deviates from planarity as seen in a twist between the oxime and amide groups about the C8—C9 bond; the O1-C8-C9-N4 torsion angle is -164.0 (4)°. The flattened geometry of molecule results in the appearance of short intramolecular contacts H10···O2 is 2.34 Å and H7C···H3N 2.28 Å. The C—N bond distance in the azomethine group is 1.277 (4) Å, and the N2—C6—C1 angle is 115.7 (3)°. The pyridine-N atom is situated in an *anti*- position with respect to the azomethine group. Finally, the C—N and C—C bond lengths within the pyridine ring are normal for 2-substituted pyridine derivatives (Krämer *et al.*, 2002; Kovbasyuk *et al.*, 2004).

In the crystal packing molecules are united by  O2—H···O1 hydrogen bonds, Table 1, where oximic-oxygen atom acts as donor and the hydrazone-oxygen atom acts as an acceptor (Fig. 2). This interaction probably results in the elongation of the C8—O1 bond to 1.233 (4) Å as compared with its mean value 1.210 Å (Bürgi & Dunitz, 1994). Due to the presence of the O2—H···O1 hydrogen bonds, zig-zag supramolecular chains are formed along the *c* axis.

### Experimental

Compound (I) was prepared according to the reported procedure (Moroz *et al.*, 2008*b*).

### Refinement

All H atoms were observed in a difference Fourier map, but C—H hydrogen atoms were placed at calculated positions and treated as riding on their parent atoms [C—H = 0.93-0.96 Å and *U*_iso_(H) = 1.2- 1.5*U*_eq_(C)]. The N—H and O—H hydrogen atoms were fully refined; O-H = 0.84 (5) Å and N-H = 0.87 (4) Å. In the absence of significant anomalous scattering effects, 766 Friedel pairs were averaged in the final refinement.

### Crystal data

**Table:**

C_10_H_12_N_4_O_2_	*F*(000) = 464
*M**_r_* = 220.24	*D*_x_ = 1.325 Mg m^−^^3^
Monoclinic, *C**c*	Mo *K*α radiation, λ = 0.71073 Å
Hall symbol: C -2yc	Cell parameters from 5860 reflections
*a* = 4.4498 (11) Å	θ = 3.6–32.0°
*b* = 22.833 (7) Å	µ = 0.10 mm^−^^1^
*c* = 10.955 (3) Å	*T* = 293 K
β = 97.47 (2)°	Needles, white
*V* = 1103.7 (5) Å^3^	0.15 × 0.10 × 0.05 mm
*Z* = 4	

### Data collection

**Table:**

Oxford Diffraction KM-4/Xcalibur diffractometer with a Sapphire3 detector	958 independent reflections
Radiation source: Enhance (Mo) X-ray Source	793 reflections with *I* > 2σ(*I*)
graphite	*R*_int_ = 0.059
Detector resolution: 16.1827 pixels mm^-1^	θ_max_ = 25.0°, θ_min_ = 3.6°
φ scans and ω scans with κ offset	*h* = −5→5
Absorption correction: multi-scan (*CrysAlis CCD*; Oxford Diffraction, 2006)	*k* = −25→26
*T*_min_ = 0.986, *T*_max_ = 0.995	*l* = −12→12
3899 measured reflections	

### Refinement

**Table:**

Refinement on *F*^2^	Secondary atom site location: difference Fourier map
Least-squares matrix: full	Hydrogen site location: inferred from neighbouring sites
*R*[*F*^2^ > 2σ(*F*^2^)] = 0.046	H atoms treated by a mixture of independent and constrained refinement
*wR*(*F*^2^) = 0.097	*w* = 1/[σ^2^(*F*_o_^2^) + (0.0453*P*)^2^ + 0.2337*P*] where *P* = (*F*_o_^2^ + 2*F*_c_^2^)/3
*S* = 1.10	(Δ/σ)_max_ < 0.001
958 reflections	Δρ_max_ = 0.13 e Å^−^^3^
155 parameters	Δρ_min_ = −0.16 e Å^−^^3^
2 restraints	Absolute structure: nd
Primary atom site location: structure-invariant direct methods	

### Special details

**Table:**

Geometry. All e.s.d.'s (except the e.s.d. in the dihedral angle between two l.s. planes) are estimated using the full covariance matrix. The cell e.s.d.'s are taken into account individually in the estimation of e.s.d.'s in distances, angles and torsion angles; correlations between e.s.d.'s in cell parameters are only used when they are defined by crystal symmetry. An approximate (isotropic) treatment of cell e.s.d.'s is used for estimating e.s.d.'s involving l.s. planes.
Refinement. Refinement of *F*^2^ against ALL reflections. The weighted *R*-factor *wR* and goodness of fit *S* are based on *F*^2^, conventional *R*-factors *R* are based on *F*, with *F* set to zero for negative *F*^2^. The threshold expression of *F*^2^ > σ(*F*^2^) is used only for calculating *R*-factors(gt) *etc*. and is not relevant to the choice of reflections for refinement. *R*-factors based on *F*^2^ are statistically about twice as large as those based on *F*, and *R*- factors based on ALL data will be even larger.

### Fractional atomic coordinates and isotropic or equivalent isotropic displacement parameters (Å^2^)

**Table:**

	*x*	*y*	*z*	*U*_iso_*/*U*_eq_	
N1	−0.4385 (8)	0.29326 (15)	0.1660 (4)	0.0601 (10)	
N2	−0.0269 (7)	0.41668 (13)	0.0900 (3)	0.0411 (8)	
N3	0.1840 (7)	0.45542 (13)	0.1431 (3)	0.0418 (8)	
N4	0.6418 (7)	0.52027 (13)	0.2434 (3)	0.0402 (8)	
O1	0.1686 (7)	0.50958 (13)	−0.0301 (3)	0.0624 (9)	
O2	0.8544 (6)	0.55993 (14)	0.2979 (3)	0.0559 (8)	
C1	−0.3464 (9)	0.33542 (16)	0.0958 (4)	0.0439 (10)	
C2	−0.4547 (11)	0.3399 (2)	−0.0272 (4)	0.0624 (13)	
H2	−0.3891	0.3701	−0.0743	0.075*	
C3	−0.6589 (11)	0.2998 (2)	−0.0793 (5)	0.0763 (16)	
H3	−0.7310	0.3019	−0.1628	0.092*	
C4	−0.7553 (11)	0.2573 (2)	−0.0090 (5)	0.0706 (15)	
H4	−0.8962	0.2296	−0.0423	0.085*	
C5	−0.6403 (13)	0.2558 (2)	0.1128 (6)	0.0750 (15)	
H5	−0.7085	0.2265	0.1614	0.090*	
C6	−0.1176 (8)	0.37676 (17)	0.1582 (3)	0.0425 (10)	
C7	−0.0053 (11)	0.3692 (2)	0.2922 (4)	0.0607 (13)	
H7A	−0.0416	0.4044	0.3357	0.091*	
H7B	0.2082	0.3611	0.3022	0.091*	
H7C	−0.1103	0.3372	0.3246	0.091*	
C8	0.2702 (8)	0.50043 (16)	0.0783 (3)	0.0405 (9)	
C9	0.4981 (9)	0.54051 (15)	0.1430 (3)	0.0404 (10)	
C10	0.5493 (13)	0.59805 (19)	0.0889 (5)	0.0755 (16)	
H10A	0.3634	0.6119	0.0440	0.113*	
H10B	0.7001	0.5945	0.0342	0.113*	
H10C	0.6177	0.6253	0.1534	0.113*	
H3NA	0.239 (8)	0.4565 (15)	0.222 (4)	0.035 (10)*	
H2OA	0.932 (11)	0.5383 (19)	0.355 (5)	0.061 (14)*	

### Atomic displacement parameters (Å^2^)

**Table:**

	*U*^11^	*U*^22^	*U*^33^	*U*^12^	*U*^13^	*U*^23^
N1	0.063 (2)	0.045 (2)	0.069 (3)	−0.013 (2)	−0.0028 (19)	0.0015 (19)
N2	0.0370 (18)	0.0396 (16)	0.0439 (18)	−0.0042 (15)	−0.0056 (14)	−0.0027 (15)
N3	0.0436 (19)	0.0436 (19)	0.0353 (19)	−0.0083 (16)	−0.0062 (15)	−0.0009 (14)
N4	0.0361 (18)	0.0403 (17)	0.0408 (18)	−0.0022 (15)	−0.0078 (14)	−0.0055 (14)
O1	0.071 (2)	0.0603 (18)	0.0478 (17)	−0.0208 (16)	−0.0238 (15)	0.0105 (14)
O2	0.0555 (18)	0.0570 (18)	0.0478 (17)	−0.0045 (15)	−0.0220 (13)	−0.0013 (14)
C1	0.036 (2)	0.041 (2)	0.054 (2)	0.0004 (18)	0.0032 (19)	−0.0047 (18)
C2	0.068 (3)	0.071 (3)	0.046 (2)	−0.025 (3)	0.002 (2)	−0.007 (2)
C3	0.072 (4)	0.089 (4)	0.063 (3)	−0.026 (3)	−0.007 (3)	−0.020 (3)
C4	0.051 (3)	0.058 (3)	0.100 (4)	−0.020 (2)	0.000 (3)	−0.028 (3)
C5	0.078 (4)	0.052 (3)	0.093 (4)	−0.028 (3)	0.004 (3)	−0.003 (3)
C6	0.045 (3)	0.038 (2)	0.043 (2)	0.0000 (18)	0.0022 (19)	−0.0043 (17)
C7	0.075 (3)	0.061 (3)	0.044 (2)	−0.017 (2)	−0.005 (2)	−0.0039 (19)
C8	0.040 (2)	0.038 (2)	0.039 (2)	0.0029 (18)	−0.0085 (17)	0.0004 (17)
C9	0.047 (3)	0.0368 (19)	0.0342 (19)	0.0021 (18)	−0.0066 (17)	−0.0001 (16)
C10	0.087 (4)	0.059 (3)	0.069 (3)	−0.024 (3)	−0.034 (3)	0.015 (3)

### Geometric parameters (Å, °)

**Table:**

N1—C5	1.320 (6)	C3—C4	1.345 (7)
N1—C1	1.330 (5)	C3—H3	0.9300
N2—C6	1.277 (4)	C4—C5	1.366 (8)
N2—N3	1.364 (4)	C4—H4	0.9300
N3—C8	1.334 (5)	C5—H5	0.9300
N3—H3NA	0.87 (4)	C6—C7	1.498 (5)
N4—C9	1.285 (5)	C7—H7A	0.9600
N4—O2	1.388 (4)	C7—H7B	0.9600
O1—C8	1.233 (4)	C7—H7C	0.9600
O2—H2OA	0.84 (5)	C8—C9	1.477 (5)
C1—C2	1.374 (6)	C9—C10	1.471 (5)
C1—C6	1.488 (5)	C10—H10A	0.9600
C2—C3	1.362 (6)	C10—H10B	0.9600
C2—H2	0.9300	C10—H10C	0.9600
			
C5—N1—C1	117.2 (4)	N2—C6—C1	115.7 (3)
C6—N2—N3	117.7 (3)	N2—C6—C7	124.4 (4)
C8—N3—N2	120.1 (3)	C1—C6—C7	119.9 (4)
C8—N3—H3NA	116 (2)	C6—C7—H7A	109.5
N2—N3—H3NA	122 (2)	C6—C7—H7B	109.5
C9—N4—O2	111.7 (3)	H7A—C7—H7B	109.5
N4—O2—H2OA	97 (3)	C6—C7—H7C	109.5
N1—C1—C2	121.7 (4)	H7A—C7—H7C	109.5
N1—C1—C6	115.9 (3)	H7B—C7—H7C	109.5
C2—C1—C6	122.4 (4)	O1—C8—N3	123.3 (4)
C3—C2—C1	119.4 (5)	O1—C8—C9	120.0 (3)
C3—C2—H2	120.3	N3—C8—C9	116.7 (3)
C1—C2—H2	120.3	N4—C9—C10	125.5 (4)
C4—C3—C2	119.3 (5)	N4—C9—C8	115.0 (3)
C4—C3—H3	120.3	C10—C9—C8	119.5 (3)
C2—C3—H3	120.3	C9—C10—H10A	109.5
C3—C4—C5	118.1 (4)	C9—C10—H10B	109.5
C3—C4—H4	121.0	H10A—C10—H10B	109.5
C5—C4—H4	121.0	C9—C10—H10C	109.5
N1—C5—C4	124.2 (5)	H10A—C10—H10C	109.5
N1—C5—H5	117.9	H10B—C10—H10C	109.5
C4—C5—H5	117.9		
			
C6—N2—N3—C8	−175.2 (3)	C2—C1—C6—N2	−0.3 (6)
C5—N1—C1—C2	−0.3 (6)	N1—C1—C6—C7	0.2 (5)
C5—N1—C1—C6	−179.7 (4)	C2—C1—C6—C7	−179.2 (4)
N1—C1—C2—C3	−0.9 (7)	N2—N3—C8—O1	−0.1 (6)
C6—C1—C2—C3	178.5 (4)	N2—N3—C8—C9	179.6 (3)
C1—C2—C3—C4	1.3 (8)	O2—N4—C9—C10	0.7 (6)
C2—C3—C4—C5	−0.7 (8)	O2—N4—C9—C8	179.1 (3)
C1—N1—C5—C4	0.9 (8)	O1—C8—C9—N4	−164.0 (4)
C3—C4—C5—N1	−0.5 (9)	N3—C8—C9—N4	16.3 (5)
N3—N2—C6—C1	−179.7 (3)	O1—C8—C9—C10	14.5 (6)
N3—N2—C6—C7	−0.8 (5)	N3—C8—C9—C10	−165.2 (4)
N1—C1—C6—N2	179.1 (3)		

### Hydrogen-bond geometry (Å, °)

**Table:**

*D*—H···*A*	*D*—H	H···*A*	*D*···*A*	*D*—H···*A*
O2—H2OA···O1^i^	0.84 (5)	1.88 (5)	2.709 (4)	170 (5)
N3—H3NA···N4	0.87 (4)	2.30 (4)	2.640 (4)	104 (3)

Symmetry codes: (i) *x*+1, −*y*+1, *z*+1/2.
